# FREQUENCY AND RISK FACTORS OF CLAVICLE FRACTURES IN PROFESSIONAL CYCLISTS

**DOI:** 10.1590/1413-785220162405157391

**Published:** 2016

**Authors:** ALEXANDRE YUKIO NISHIMI, PAULO SANTORO BELANGERO, RAFAEL DE SOUZA MESQUITA, CARLOS VICENTE ANDREOLI, ALBERTO DE CASTRO POCHINI, BENNO EJNISMAN

**Affiliations:** 1. Universidade Federal de São Paulo (UNIFESP), Escola Paulista de Medicina, Department of Orthopedics and Traumatology, São Paulo, SP, Brazil.

**Keywords:** Fractures, bone. Clavicle. Athletic injuries. Bicycling. Risk factors.

## Abstract

**Objective:**

To evaluate the prevalence of clavicle fractures in professional and amateur cyclists and evaluate the factors associated with its occurrence.

**Method:**

One hundred and forty professional and amateur athletes were interviewed through a questionnaire regarding age and time practicing bicycling, among others.

**Results:**

Among the 140 evaluated cyclists, there were 19 (13.5%) clavicle fractures associated with this sports modality.

**Conclusion:**

There was a positive association between time practicing bicycling and frequency of clavicle fractures, as well as between hours of weekly training and clavicle fractures. Level of Evidence IV, Case-Series.

## INTRODUCTION

Cycling is a sport that has been growing in Brazil and worldwide. With increased cycling practice, there was an increase of certain injuries. In the same period in the UK, the total number of deaths registered among cyclists increased by 12% and the number of serious casualties increased by 16%, from 2,660 to 3,085.^1^

Cyclists evaluated between 2003 and 2009 in Spain had twice the risk of traumatic injuries as compared to cyclists evaluated between 1980 and 1990. Apart from these traumatic injuries, cyclists had prevalent tendinopathy (41.5%) and muscle injuries (56.6%).^2^

Between 2009 and 2010, clavicle and shoulder fractures were among the most frequent (16%). Collisions between cyclists are the most frequent cause of accidents (39%), followed by collisions with obstacles, loss of control, and fall, among others.^3^

Age is one of the predisposing factors for injuries involving cycling; adolescents and young adults were those at greatest risk of accidents. The group of cyclists between 16 and 29 years old had higher risks of accidents per kilometer, as compared to other age groups; moreover, this group showed the highest prevalence of deaths and serious accidents.^4^ Clavicle fractures implicate in withdrawal from training, and it is associated with long recovery periods to reach the pre-fracture performance.

The study hypothesis is that cyclists have a higher prevalence of clavicle fractures, as compared to the general population. Thus, the aim of this study was to evaluate the prevalence of clavicular fractures in professional and amateur cyclists and to assess the factors associated to their occurrence. 

## METHODOLOGY

Participants were randomly selected and previously agreed to participate in the survey, which was approved by the Research Ethics Committee, under protocol number 402004. The study was conducted from October 2012 to February 2013.

One hundred and forty professional and amateur athletes were interviewed using a questionnaire containing multiple choice and essay questions to be responded by the participants after direct instructions of the interviewer (RM). The sample was considered a convenience sample according to the availability of athletes.

The study included professional and amateur adult cyclists; respondents who practiced only for leisure or hobby were excluded from the sample (exclusion criteria considered below level 5 of Tegner's physical activity scale).

The questionnaire included questions related to age, sport modality, practice time, training frequency, professionalism and sponsorship in sports practice, number of races, injuries suffered in the previous year, clavicle fracture or other injuries, type of treatment, and method and recovery time to return to sports practice at the same level pre-injury. (Annex 1) 

### Statistical Analysis

All the results were statistically analyzed using SPSS for Windows (SPSS Inc, Chicago, IL, USA); absolute and relative frequencies for qualitative variables were calculated. p-Values lower than 0.05 were considered statistically significant.

## RESULTS

**Figure 1:**
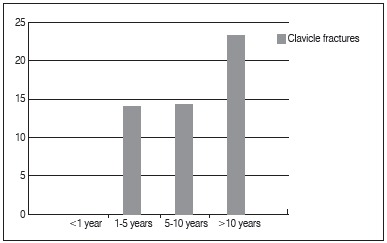
Clavicle fractures and years practicing cycling.

Among the 140 cyclists assessed, 19 (13.5%) had clavicle fractures associated to cycling; there was a statistically significant association between cycling practice time and frequency of clavicle fractures. Of the 25 athletes who started to practice cycling for less than one year, none of them suffered any clavicle fractures. However, among cyclists with over 10 years of practice, 23.3% (7/30) had fractured their clavicle, as shown in Figure 1.

**Figura 2:**
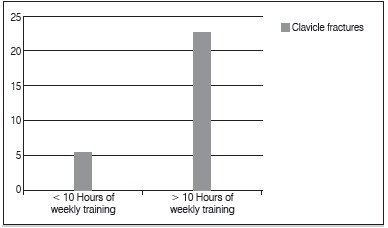
Clavicle fractures and hours of weekly training.

Moreover, there were a correlation between weekly hours training and clavicle fractures. Of the 66 cyclists who trained more than 10h per week 22.7% had fractured their clavicle while cycling, as shown in Figure 2. There was no statistical correlation between the number of cycling races attended in the previous year and clavicle fractures, as well as the age of practitioners and the frequency of clavicle fractures. (Table 1)

**Table 1:** Presence of clavicular injuries among cyclists and their associated factors.


	Clavicular injuries					
Variables	No		Yes		Total	***p***
	N	%	N	%		
Age (years old)						0.345
Up to 30	36	87.8	5	12.2	41	
31 a 40	54	90.0	6	10.0	60	
41 a 50	23	76.7	7	23.3	30	
> 50	8	88.9	1	11.1	9	
Time practicing cycling (years)						0.023
< 1	25	100.0	0	0.0	25	
1 a 5	49	86.0	8	14.0	57	
5 a 10	24	85.7	4	14.3	28	
> 10	23	76.7	7	23.3	30	
Hours per week						0.019
< 5	17	85.0	3	15.0	20	
5 a 10	53	98.1	1	1.9	54	
> 10	51	77.3	15	22.7	66	
Number of races						0.764
< 5	89	86.4	14	13.6	103	
5 a 10	19	95.0	1	5.0	20	
> 10	12	75.0	4	25.0	16	
Total	121	86.4	19	13.6	140	

## DISCUSSION

Cycling is a very popular sport globally, and has been increasingly spreading in Brazil. Professional and amateur athletes face long hours of weekly training and consequently, much time occupationally exposed to traumatic injuries.

Several international studies show clavicle fractures as the most frequent injuries in this population, and clavicle fractures are the most common bone injury,^5^ accounting for 44.4% of all fractures between 2004 and 2008, according to an American study.^6^ However, we did not find reports in our midst on the epidemiology of clavicular fractures in cycling athletes.

This study showed a 13.5% frequency of clavicle fractures in the study population against 2-6% of the general population.^7^ These fractures were responsible for, on average, five months withdrawal from sports practice.

Another important factor observed was the increase in the frequency of clavicle fractures among cyclists who practiced this sport for longer periods, as well as an association with higher number of weekly training hours. This fact shows a direct relationship between exposure time and frequency of clavicle fractures in this population. In our sample, 4 of 19 patients with clavicle fractures (21%) underwent surgical treatment.

We understand that with the increasing number of practitioners and the professionalization of this sport, the number of cyclists' clavicular fractures in Brazil is expected to increase in the coming years.

Our study has some limitations, such as the relatively small sample size and the fact we only studied road cyclists. Nevertheless, we believe that our sample is representative and may serve as a paradigm for a larger study. Additionally, it is a pioneering study in Brazil and points to an extremely relevant and current condition, which converges with increasing frequency of sports activities in our midst. Regarding practical applications, this study may be useful to support sports injuries preventive programs, which may result in sports practice interruption, particularly for high-performance athletes.

## CONCLUSION

Road cyclists show a higher frequency of clavicle fractures as compared to the general population. There is a direct relationship between time of sports practice and frequency of clavicle fractures.
